# Microbial epidemiology and clinical risk factors of carbapenemase-producing Enterobacterales amongst Irish patients from first detection in 2009 until 2020

**DOI:** 10.1016/j.infpip.2022.100230

**Published:** 2022-07-13

**Authors:** N.H. O'Connell, S. Gasior, B. Slevin, L. Power, S. Barrett, S.I. Bhutta, B. Minihan, J. Powell, C.P. Dunne

**Affiliations:** a Department of Clinical Microbiology University Limerick Hospital Group (ULHG), Limerick, Ireland; b Centre for Interventions in Infection, Inflammation & Immunity (4i), University of Limerick, Limerick, Ireland; c School of Medicine, University of Limerick, Limerick, Ireland; d Department of Infection Prevention and Control, ULHG, Limerick, Ireland; e Department of Pharmacy, ULHG, Limerick, Ireland; f Department of Gastroenterology, ULHG, Limerick, Ireland

**Keywords:** CPE Risk factors epidemiology clinical outcomes

## Abstract

**Background:**

Carbapenemase producing Enterobacterales (CPE) are major public health threats.

**Aim:**

To review microbial epidemiology of CPE, as well as clinical risk factors and infections, amongst CPE positive patients over 12 years in an Irish tertiary hospital.

**Methods:**

Retrospective observational study of data extracted from a laboratory CPE database, electronic healthcare records and manual review of patient charts. Common risk factors, treatment regimens for all CPE related infections, and clinical outcomes were ascertained.

**Findings:**

Among CPE strains isolated from 460 patients, *Klebsiella pneumoniae* carbapenemase (KPC) was the carbapenemase most frequently detected, accounting for 87.4% (459) of all CPE enzymes. Citrobacter species 177 (33.7%) were the most common species harbouring this enzyme. 428 CPE positive patients (93%) were identified in the acute hospital setting; the most common risk factor for CPE acquisition was history of hospitalisation, observed in 305 (66%) cases. Thirty patients (6.5%) had confirmed infections post-acquisition, of which four were bloodstream infections. There were 19 subsequent episodes of non CPE-related bacteraemia in this cohort. All causal mortality at 30 days was 41 patients (8.9%). However, clinical review determined that CPE was an indirect associative factor in 8 patient deaths.

**Conclusions:**

In this tertiary hospital setting, microbial epidemiology is changing; with both OXA-48 enzymes and KPC-producing Citrobacter species becoming more prevalent. Whilst the burden of CPE related infections, especially bacteraemia, was low over the study period, it remains critical that basic infection prevention and control practices are adhered to lest the observed changes in epidemiology result in an increase in clinical manifestations.

## Introduction

Carbapenemase producing Enterobacterales (CPE) are a major public health concern and have spread rapidly across the globe over the past two decades [1]. They can cause serious healthcare-associated infections, which are difficult to treat due to limited treatment options associated with their high level of resistance to most antimicrobials. CPE are classified according to their differing carbapenemases. Notably, the most common enzymes being *Klebsiella pneumoniae* carbapenemase (KPC), New-Delhi Metallo-β-lactamase (NDM), oxacillinase (OXA), and Verona Integron-Mediated Metallo-β-lactamase (VIM).

As CPE incidences developed, oftentimes infections were treated with tigecycline, polymyxins and fosfomycin before the advent of newer agents such as ceftazidime/avibactam, meropenem/vaborbactam and cefiderocol. However, resistance to these newer options has been described [2,3]. Similarly, transmission is difficult to control due to clonal spread and horizontal gene transfer and thus multimodal infection prevention and control strategies have evolved with an emphasis on screening and surveillance, contact precautions and environmental cleaning to reduce spread of these pathogens [4].

The first case of CPE in Ireland was reported in 2009 at University Hospital Limerick (UHL) [5] and since then multiple outbreaks of CPE have been reported at that location, illustrating the high transmissibility of these organisms [[6], [7], [8]]. CPE became notifiable in Ireland in 2011, followed by establishment of the National Carbapenemase Producing Enterobacterales Reference Laboratory Service (NCPERLS) in 2012. Subsequently, the burden of CPE detections nationally has increased exponentially as a consequence of referral of suspect isolates for confirmation. OXA-48 has been the predominant enzyme identified in Ireland since 2013 [9]. Foley *et al.* [10] recently described their hospital's nine-year infection and control response to CPE and advise that despite intensive efforts, outbreaks of OXA-48 continue to emerge.

The global epidemiology of CPE is geographically diverse with different enzymes prevalent depending on country, and regionally within individual countries. KPC-producing CPE are the most common in the United States, Israel and certain European countries including Italy, Portugal and Greece. MBL-producing CPE have been most commonly associated with the Indian Subcontinent as well as with specific countries in Europe, including Romania, Denmark, Spain, and Hungary. The epicenters of OXA-48-like-producing CPE are Turkey and other Mediterranean countries, North Africa and other European countries (France, Germany, Spain, and Belgium. [[11], [12], [13], [14]].

There are varied reports on clinical risk factors for CPE acquisition, colonisation *vs.* infection and mortality rates. One of the largest single centre studies on this topic, published in 2020 [15], concluded that long inpatient stays, exposure to carbapenems amongst other antimicrobials, dialysis, mechanical ventilation, transfusion and complex thoracic pathology were the risk factors associated with KPC gene acquisition, but exposure to other KPC colonised patients did not play a major role. A 2019 Spanish study [16] reviewed all OXA-48 infections from October 2014 to December 2016 and showed 30-day and 90-day mortality rates of 8.3% and 20.8 %, respectively. Following the rapid increase of CPE infections in Italy, collated by national surveillance of CPE bloodstream infections in 2013, all 7,632 CPE-BSI cases in the years 2014–17 were studied [17]. Seventeen percent of patients (1,165/6,869) were reported to have died at the time of publishing those data. Mortality was associated significantly with age (*P* < 0.01); in particular, mortality was higher in children 0–9 years (5/31, 16.1%) and in the elderly aged ≥ 75 years (528/2,428, 21.7%). A multicentre observational study in 11 hospitals from 7 Latin American countries [18] determined that CPE infection is an independent mortality predictor associated with in-hospital mortality. Whilst most studies report all-cause mortality rates, there is limited information on actual direct or indirect association with death.

The aim of this study is to review the microbial epidemiology and clinical risk factors amongst CPE positive patients in a tertiary hospital providing services to the mid-west of Ireland from 2009 to 2020, with an emphasis on subsequent CPE infections, their clinical management and mortality outcomes. A secondary aim was to review any non-CPE associated bloodstream infections in this cohort and their empiric treatment and mortality outcomes.

## Methods

### Ethical approval

This study was approved by the Research Ethics Committee of University Limerick Hospital Group, Limerick, Ireland.

### Setting

The Department of Clinical Microbiology at UHL provides a centralised microbiology service for six acute hospital sites known as the University of Limerick Hospitals Group (ULHG). The bed complement has increased over the study timeframe to 850 beds; population circa 400,000 people. Of note, there are no electronic patient records in this group of hospitals. There have been multiple outbreaks associated with resistant bacteria across the group of hospitals as previously described, which demonstrates the infrastructural challenges encountered with ward design (lack of single rooms) and overcrowding [19,20]. Infection Prevention and Control (IPC) resources have also improved over the timescale of this study with an increase in nursing resources from 2 whole time equivalents (WTEs) for all IPC duties to 10 WTEs, which comprise, since 2019, a dedicated CPE team lead by a consultant microbiologist, an assistant director of nursing for CPE control and 2 IPC staff nurses. Resources have also been allocated by the Irish government to clinical microbiology departments nationally to enable implementation of the Health Service Executive (HSE) CPE screening program [21]; including 2 medical scientists recruited for CPE diagnostics. Infection control measures have been in place at UHL since the first outbreak in 2011 [6] and are guided by best international and national guidance [4,22]. They involve strict contact precautions; single en-suite room when feasible but otherwise single room with a poster alert on the door signalling the transmission-based precautions to be adopted for the patient accommodated therein; dedicated and or single use equipment; identification and screening of close contacts; dissemination of factsheets on CPE to patients; IPC alert generated on both the healthcare patient administration system (iPMS, eHealth Ireland) and the clinical surveillance software (ICNet, Baxter Healthcare, Illinois, USA); communication to General Practitioners (and other healthcare premises where applicable); and finally terminal disinfection on discharge.

Operational outbreak team meetings are convened at weekly intervals enabling timely surveillance to identify and manage clusters and/or outbreaks. The UHL site has had multiple outbreaks in multi-bedded wards since 2014 [6,7]. A cohort ward was designated for managing CPE positive patients in 2015 at UHL and although it has limited single rooms (5 in total), there are small multi-bedded rooms for the accommodation of CPE positive patients who are colonised with similar CPE enzymes. A strategic CPE committee meeting is held every 2 months to review CPE surveillance data, CPE admissions and their bed management, screening compliance and audit results.

### Microbiological and molecular detection of CPE

Screening for CPE, as cited previously, is in accordance with the Irish HSE and national Health Protection Surveillance Centre (HPSC) recommendations [21]. Patients in the hospital group are screened when transferred between ULHG sites and other hospitals, long-term care facilities or nursing homes in Ireland; or have a history of an acute admission in the past 12 months to any hospital within our hospital group; or have any history of being hospitalised abroad. In addition, haemodialysis patients are screened every three months. Patients in ICU and HDU are screened on admission and have weekly screens thereafter until discharge from these units. Furthermore, additional screening is conducted when an outbreak is declared on a ward.

Initially, the Centers for Disease Control and Prevention (CDC) method [23] was adopted for CPE detection before the introduction of selective agar. CHROMagar™ KPC was used from 2012 to 2018 and CHROMagar™ mSuperCarba from 2018 onwards; both CHROMagar Company, Paris, France. MALDI-TOF MS (Bruker Diagnostics) identification is performed on all colonies from these culture methods, as previously described [24] and non-Enterobacterales are disregarded. Antimicrobial susceptibility testing is performed using broth microdilution (ARIS Sensititre® system (Thermo Fisher Scientific Inc., Massachusetts, USA)). Elevated carbapenem minimum inhibitory concentrations (MICs) for meropenem and ertapenem are confirmed by E-test (AB Biodisk, Solna, Sweden) following EUCAST guidelines [25]; ertapenem resistance being determined by MIC >1mmol/L, meropenem resistance MIC >8mmol/L. Isolates with elevated carbapenem MICs were further evaluated up to 2014 by using the modified Hodge test [26]. Commercially available diagnostic kits (Rosco Diagnostica A/S, Taastrip, Denmark) consisting of meropenem discs supplemented with β-lactamase inhibitors (meropenem + dipicolinic acid, meropenem + boronic acid and meropenem + cloxacillin) are used to phenotypically distinguish CPE isolates. Since 2014, suspect colonies on chromogenic agar are tested for the presence of a CPE gene using the Carba-R GeneXpert® System, Cepheid, California, USA. Prior to the establishment of the National Carbapenemase Producing Enterobacterales Reference Laboratory Service (CPERL) at University Hospital Galway, Galway, Ireland in 2013, all suspect isolates were sent to Antimicrobial Resistance and Healthcare Associated Infections (AMRHAI) reference unit, Public Health England, Colindale, London for CPE confirmation by molecular methods.

### Study time frame

A retrospective observational study was undertaken with data compiled on both CPE isolates detected and the patients harbouring them, at ULHG microbiology department, for the period between January 1^st^ 2009 and December 31^st^ 2020.

### Identifying CPE positive cases

Data were extracted from a designated CPE database within the microbiology department, which records positive CPE results from all clinical specimens; both routine clinical samples and screening swabs. Each CPE positive patient was recorded once only during this study, subsequent detections of different organisms and/or genes were appended to the patient record. Demographic, clinical characteristics and inpatient location data were collected from a manual patient chart review and from electronic databases including the healthcare patient administration system (iPMS, eHealth Ireland) clinical surveillance software (ICNet, Baxter Healthcare, Illinois, USA), radiological imaging requests (National Integrated Medical Imaging System (NIMIS), eHealth Ireland) as well as clinical notes, inputted by the microbiology medical team, from the Laboratory Information Management System (iLab, Dedalus Healthcare, Milan, Italy). All patient data were anonymised in compliance with the General Data Protection Regulation (GDPR). Common risk factors for acquisition were collated including previous healthcare attendance, domicile in nursing or long-term care setting, CPE contact status, recent surgery (ascertained by data extraction from Hospital Inpatient Enquiry system (HIPE) coding) or endoscopy (extracted data from central endoscopy database) within preceding 3 month prior to detection, recent carbapenem exposure, and length of stay. CPE colonisation or infection status and relevant microbiological treatment for eligible patients were determined with CPE infections studied for inpatients only. Infections were delineated as follows- CPE isolated from blood cultures or from other clinical specimens (sputum, soft tissue, intra-operative fluids/swabs) wherein clinical correlation was consistent with an infection as defined by the presence of symptoms, signs and imaging in accordance with definitions adopted from previous European healthcare associated point prevalence surveys [27]. A urine culture was considered to represent infection if culture was positive with >/= 10^5^ colony forming units (CFU)/mL urine in patients exhibiting symptoms and signs of urinary tract infection. Determination of the contribution of CPE status to mortality was determined for hospitalised patients who died during their admission, as previously described by two researchers [28], by accessing the National Data Registry with correlation of chart review findings of last hospital admissions with both microbiological and clinical data near the time of death and death certification where available.

## Results

### Phenotypic characteristics

**Figure 1:**
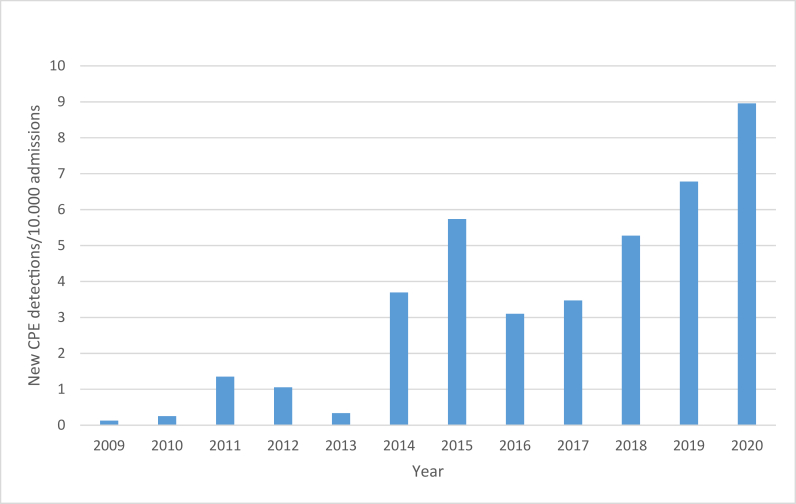
Rate of new CPE case detections/10,000 admissions.

From January 2009 to December 2020, there were 513 different Enterobacterales isolates identified that produced carbapenemases, from 460 patients and a total of 118,319 screening specimens in the clinical microbiology laboratory in ULHG. There was an annual exponential increase in numbers of enzymes detected since 2016 (see Table I). Likewise, the rate of new case detection per 10,000 admissions increased from 0.127 in 2009 to 8.957 in 2020 (see Figure 1). 417 patients (91%) had a single CPE isolate detected, 33 (7%) had two isolates and 10 (2%) patients had three isolates, see Table I. Just three patients in this period had more than one carbapenemase type detected; all from different organisms (i.e., no single isolate has exhibited more than one carbapenemase). The types of enzymes commonly detected within the timeframe of this study were KPC, accounting for (459) 87.4% of all CPE detections, with OXA-48 and NDM detections accounting for (41) 7.8% and (23) 4.3%, respectively. Only 2 patient isolates were associated with an IMI enzyme, see Table II.

**Table I:** Number of detections of patients with isolates encoding CPE enzyme(s) per annum 2009-2020

	Number of CPE genes			
Year	1	2	3	No. of patients
2009	1		1	2
2010	4			4
2011	11			11
2012	10			10
2013	8			8
2014	39	3	2	44
2015	53	7		60
2016	36			36
2017	42	3		45
2018	58	3	2	63
2019	69	8	3	80
2020	86	9	2	97
**Total**	**417**	**33**	**10**	**460**

**Table II:** Carbapenemases produced by isolated bacterial species

Species	IMI	KPC	NDM	OXA-48	Total
*Citrobacter* sp.		177			177
*K. pneumoniae*		116	13	11	140
*E. coli*		42	9	22	73
*Enterobacter* sp.	2	57	1	2	62
*K. oxytoca*		44		4	48
Other Klebsiella sp.		7			7
Other		5		2	7
No ID		11			11
**Total**	**2**	**459**	**23**	**41**	**525**

Furthermore, the first detection of OXA-48 in our cohort occurred in 2014. It accounted for 2.3% of CPE detections in that year, with OXA-48 detections increasing steadily between 2014 to 2018 (to a high of 12.7% of detections) and in 2020 accounted for 7.2% of overall CPE detections. The species positive for CPE gene detection were diverse (see Table III). In the earlier years of this study, *Klebsiella pneumoniae* was the predominant CPE positive organism, however CPE-producing *Citrobacter* species have become more prevalent since 2016. Overall, *K. pneumoniae* and *Citrobacter* species accounted for 26.6% (n = 140) and 33.7% (n = 177), respectively, of all CPE positive Enterobacterales. *Escherichia coli* was the most frequently isolated Enterobacterale positive for the OXA-48 gene (n = 22, 53.7% of OXA-48 positive isolates, see Table III).

**Table III:** Isolated bacterial species producing carbapenemases 2009-2020

Year	Citrobacter	K. pneumoniae	E. coli	Enterobacter	K. oxytoca	Other klebsiella	Other	No ID	Total
2009		2	1	1					4
2010		4							4
2011		11							11
2012		10							10
2013	2	5		1					8
2014	4	32	6	1	8		1		52
2015	14	20	8		23		1		66
2016	18	9	5	3	1		1		37
2017	17	12	7	7	3	3			49
2018	25	12	11	18	4	1		1	72
2019	39	16	19	17	4	1	2	4	102
2020	58	7	16	14	5	2	2	6	110
**Total**	**177**	**140**	**73**	**62**	**48**	**7**	**7**	**11**	**525**

**Figure 2:**
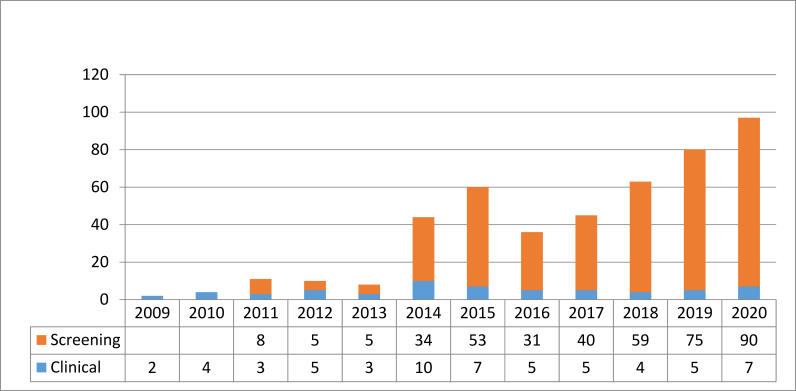
Specimen type for CPE detections (screening = rectal swab, clinical = routine specimen sent for culture (e.g. urine, sputum, pus, wound, blood).

The specimen type associated with the greatest yield of CPE detection was rectal screen, accounting for 87% of newly identified patients. The remaining specimens were: urine (39 samples), sputum (9 samples), wound (10 samples) and blood (2 samples), see Figure 2. Antimicrobial susceptibilities of those isolates tested are presented in Table IV. Interestingly *K. oxytoca* isolates were more susceptible to cotrimoxazole (97.6%) and ciprofloxacin (91.3%) by comparison to *K. pneumoniae* CPE isolates (47.9% and 36.2%, respectively).

**Table IV:** Antimicrobial susceptibilities of CPE isolates tested from 2009-2020

	AMI	CHLOR	CIP	CTZAV	ERT	FOS	GEN	LEV	MERO	TIGE	TRSM
2009	25.0%	0.0%	0.0%	0.0%	0.0%	0.0%	66.7%	0.0%	66.7%	0.0%	0.0%
2010	0.0%	0.0%	0.0%	0.0%	0.0%	100.0%	100.0%	0.0%	0.0%	50.0%	0.0%
2011	45.5%	0.0%	0.0%	0.0%	0.0%	0.0%	90.9%	0.0%	0.0%	0.0%	27.3%
2012	0.0%	0.0%	0.0%	0.0%	0.0%	0.0%	80.0%	0.0%	0.0%	42.9%	0.0%
2013	28.6%	0.0%	14.3%	0.0%	0.0%	100.0%	100.0%	16.7%	0.0%	0.0%	33.3%
2014	50.0%	48.6%	26.5%	0.0%	4.4%	89.5%	59.2%	23.8%	14.3%	87.2%	41.7%
2015	88.5%	68.9%	64.1%	0.0%	0.0%	100.0%	74.6%	66.7%	21.5%	79.7%	83.3%
2016	89.2%	34.8%	27.0%	0.0%	8.3%	100.0%	51.4%	32.1%	16.2%	55.6%	64.5%
2017	89.1%	54.8%	34.0%	100.0%	13.0%	100.0%	68.1%	48.8%	21.7%	66.7%	54.8%
2018	94.4%	58.5%	38.9%	96.8%	4.2%	93.4%	58.3%	42.3%	30.6%	65.6%	72.3%
2019	97.6%	44.6%	32.9%	96.7%	9.3%	95.7%	59.8%	27.8%	27.0%	70.6%	69.3%
2020	99.1%	55.2%	33.9%	97.2%	23.1%	93.6%	51.4%	0.0%	23.6%	64.3%	66.0%
Total	84.6%	50.9%	35.2%	97.0%	7.0%	94.2%	61.7%	42.2%	22.1%	69.7%	64.1%
No. Tested	493	411	492	263	359	312	494	237	515	277	423

Susceptibility results on all CPE isolates from 2009-2020.

ami= amikacin, chlor= chloramphenicol, cip= ciprofloxacin, ctzav= ceftazidime/avibactam, ert= ertapenem, fos= fosfomycin, gen= gentamicin, lev= levofloxacin, mero= meropenem, tige= tigecycline and trsm= cotrimoxazole.

### Patient risk factors

Of the 460 positive patients, 273 (59%) were male. The median age was 74y (IQR1 64y- IQR3 80y). There were only 4 paediatric patients, one new-born to a mother confirmed to be colonised with KPC, two children colonised with NDM (both with confirmed histories of travel to the Indian subcontinent, with one hospitalised) and one patient with a KPC positive isolate from a urine culture (with a history of hospitalisation in Italy).

Forty patients (8.7%) were nursing home residents and 17 patients (3.7%) were living in long-term care facilities. Fifty percent (n=20) of the nursing home patients also had a history of hospital admission in the prior 12 months, with 94% (n=16) of long-term care resident sharing a similar acute admission history. Overall, 305 of all CPE positive patients (66%) had a history of inpatient admission in ULHG in the preceding year. Three patients had a history of hospitalisation abroad (2 in the Indian subcontinent and one in Italy). Four hundred and twenty-eight (93%) of CPE patients were identified in the acute hospital setting. Overall, sixty-two (13.5%) of all CPE positive patients were detected on admission screening with 50 of this cohort (80%) having had a recent hospital admission. Twenty (4.3%) patients were positive upon admission screening to the Intensive Care Unit. One hundred and sixteen patients (25.2%) were listed as CPE contacts and a further thirteen patients, who had been delisted subsequently as contacts (i.e., had four negative screens taken > one week apart) also tested positive later when screened as a consequence of infection control team recommendations. For those positive patients who had a negative admission screen and were detected latterly (349, (75.9%)), their mean and median lengths of stay were 18.8 and 12 days, respectively (IQR 7–12 days). Four patients attending dialysis tested positive as part of the dialysis three monthly rolling screening program. Forty-five CPE patients were not admitted inpatients; that is, tested positive from community settings (general practice or long-term care facilities) or outpatient clinics.

Forty-five (9.8%) patients of the total cohort were exposed to a carbapenem three months before CPE was detected; twelve (26.7%) of whom were known KPC contacts. 76 (16.5%) patients had a history of an anaesthetic in theatre 3 months before CPE detection (adopted surrogate marker for surgery) and 90 (19%) had a history of endoscopy within the same timeframe before isolation of CPE. Table V illustrates associated chronic medical conditions with cardio-vascular conditions accounting for 59% (270 patients) of the case burden and 100 patients (21.7%) having three or more co-morbidities. There was almost an equal gender distribution between the various co-morbidities except for hepatic disease (60.5% male incidence).

**Table V:** Co-morbidities of CPE positive patients

Co-morbidity	Number of patients n (%)	Male n (%)
Renal	137 (30%)	69 (50.4%)
Hepatic	43 (9%)	26 (60.5%)
Pulmonary	155 (33.7%)	88 (56.8%)
Cardiovascular	279 (60.7%)	149 (53.4%)
Diabetes mellitus	109 (23.7%)	77 (70.6%)
Immuno-compromise	95 (20.7%)	44 (46.3%)
2 co-morbidities	137 (29.8%)	73 (53.2%)
>/= 3 co-morbidities	100 (21.7%)	56 (56%)

Previous colonisation with other multi-drug resistant micro-organisms (MDRO) was determined with 82 (17.8%), 59 (12.8%) and 41 (8.9%) patients positive for MRSA, VRE and ESBL-producing coliforms, respectively. Overall, 127 (27.6%) patients previously harboured one MDRO with 23 (5%) and 3 (0.7%) patients having a history of being colonised with two and three MDROs respectively.

### Associated morbidity and mortality

Thirty (6.5%) patients had a confirmed CPE infection during this timeframe; see Table VI which details the source of infection, the antimicrobial regimens used for treatment as well as clinical outcomes at 30- and 90- days post diagnosis. Only four patients had positive CPE blood cultures between 2009 and 2020; source of their bacteraemia was intra-abdominal in two patients with another associated having a complicated skin and soft tissue infection (cSSTI), and a urinary source in the fourth case. All had invasive devices *in situ*. Two (50%) of these bacteraemic patients were successfully treated for their blood-stream infections. Twenty nine (96.7%) of infections were associated with KPC producing bacteria, with *K. pneumoniae* being the offending pathogen in 16 (53.3%) of infections.

Intra-abdominal CPE infections were diagnosed in 11 patients (36.7%) and were the most common type of infection caused by CPEs (of note, nine were not associated with concomitant bacteraemia). Another six patients were treated for respiratory tract infections; one of whom had multiple recurrent episodes. Furthermore, five patients were treated for complicated skin and soft tissue infections and two patients had CPE related bone and joint infections. Only four inpatients had signs and symptoms of a urinary tract infection and were treated for this despite the high rate of first isolates detected from clinical samples, i.e., 39 urine samples (14 requested from the acute setting, 9 from general practice, 9 from residential care and 7 from ambulatory care settings).

All causal mortality at 30 and 90 days was 41 patients (8.9%) and 75 patients (16.3%) respectively. Furthermore, upon two independent reviews of clinical notes, it was determined that CPE was not directly associated with any death but, rather, was associated indirectly with mortality in 8 patients with significant co-morbidities.

There were subsequently 19 episodes of non-CPE Enterobacterales bloodstream infections noted in the study group (see Table VII for treatment regimens and mortality at 30 days). Despite being CPE colonised, empiric treatment included coamoxiclav and gentamicin (2 patients), ceftriaxone (one patient) and piperacillin/tazobactam (14 cases of whom 2 had concomitant gentamicin); these agents would not have afforded active cover against the patient's previous CPE isolate. However, 9/19 (56.2%) patients had at least one negative screen for CPE between their first isolate and the non-CPE Enterobacterales positive blood culture.

## Discussion

As a consequence of ULHG experiencing the first recorded CPE outbreak in Ireland [6], a unique position is afforded to review the epidemiology of CPE across 460 patients over 12 years, which represents the largest descriptive analysis of CPE in Ireland. Most published international studies review the microbial epidemiology of CPE over shorter timeframes with smaller cohorts of patients [[29], [30], [31]].

Incidence of CPE detections has increased exponentially over the past decade in the mid-west of Ireland in tandem with national rates [9]. Initially, ULHG was unique in the Irish healthcare setting wherein KPC enzymes predominated. However, a changing trend has emerged in recent years with increasing detections of OXA-48, the most ubiquitous enzyme detected in the majority of other Irish healthcare groups [9]. This may be accounted for by the importation of OXA-48 from other healthcare settings, mediated either by the transfer or admission of patients from the mid-west to quaternary hospitals providing specialist services (neurosurgical, transplant, plastic surgery, burns or cardiothoracic care) or, alternatively, patients seeking private healthcare in other regions as the mid-west lacks a private hospital providing unscheduled emergency care. Furthermore, the admission of foreign patients from OXA-48 endemic countries could be a factor in this changing epidemiology.

In parallel, this study describes that species harbouring CPE enzymes are diversifying with *Citrobacter* species becoming dominant in recent years; indeed, now the most common bacteria causing colonisation with KPC enzymes. It is uncertain why this phenomenon has occurred but it merits further investigation with genomic analysis as previous studies have illustrated horizontal gene transfer of KPC amongst Enterobacterales [32]. A recent epidemiological study of CPE from Israel found that the proportion of patients infected with KPC producing K. pneumoniae dropped from 100% of all CPE in the first years to 28% (37/134) in 2020 [33]. Likewise, the authors found that other Enterobacterales including Citrobacter species harbouring KPC are becoming more prevalent, coupled to an increasing diverse range of carbapenemases which may reflect the globalisation of bacteriology. Whether Citrobacter species has an evolutionary advantage with respect to survival within the healthcare environment is unknown. CPE positive *E. coli* isolates were detected infrequently prior to 2014 but since that time now account for 77 (16.7%) of the CPE bacteria studied. Invariably amongst our isolates, *E. coli* isolates carried OXA-48 genes. However, CPE enzyme encoded *E. coli* isolates have thus far not been associated with an increase in infections in ULHG.

The screening program introduced within ULHG provided the impetus for development of national screening guidelines by the Irish CPE expert committee, which was established in response to the declaration of CPE as a national public health emergency in October 2017 [34]. Screening requests have expanded almost 10-fold (from 2685 in 2011 to 24,111 in 2020) both as a consequence of these national [35] and local recommendations to control outbreaks in multi-bedded areas. As previously described, [7,8] UHL is challenged with respect to its infrastructure and patient accommodation that includes nightingale style multi-bedded bays that comprise up to 14–15 patient bed-spaces serviced by limited shared sanitary facilities. There have been multiple outbreaks on these nightingale wards since 2014, leading to CPE being labelled as endemic in UHL. An appreciation for the role of the healthcare environment, with respect to sinks and shower drains, in transmission of CPE has been widely accepted over the past decade [36,37] and, although not the subject of this review, an environmental screening program of sinks and shower drains in UHL in outbreak wards has identified colonisation with multiple CPE positive isolates suggestive of potential linkages to patient acquisition meriting further investigation.

Previous studies [29,38] have described risk factors for CPE acquisition, including prior broad-spectrum antimicrobial exposure, length of stay and interaction with other colonised/infected patients. This study does not differ in that regard. 25% of detections were amongst patients who were labelled as contacts, which illustrates the importance of their inclusion in screening programs.

It is evident that being a male nursing home resident aged over 70 years with a recent history of hospital admission, during which a carbapenem course was prescribed, might elevate risk of being CPE colonised.

Whilst less than 10% of the study cohort were exposed to a carbapenem, the selective pressures exerted by antimicrobial use in general may be an important consideration. Although carbapenems are considered a restricted group of agents and compliance (with requirement to discuss with an infection specialist) has improved in recent years, from 56% in 2019 to 87% in 2020, the lack of electronic prescribing platform impedes acquisition of an electronic surveillance system for timely antimicrobial stewardship [39].

Thirty (6.5%) CPE positive patients developed infection, with KPC predominantly the causative enzyme in 28 (93%) of cases. Invasive CPE bloodstream infection occurred in only 4 patients, with 50% survival rate. Both of these surviving patients had prior rectal colonisation with CPE lending to the prompt initiation of appropriate targeted treatment, which is consistent with the literature [16,17]. However, a recent international cross-sectional study on the clinical management of carbapenem resistant Gram negative infections concluded that treatment is far from being standardized [40]. In our case, the usage of antimicrobials to treat CPE associated infections at ULHG has evolved over the timeframe studied. *In vitro* susceptibility patterns influenced prescribing practices in earlier years as well as a “combination strategy” believed, in the absence of evidence, to improve clinical efficacy and reduce the risk of further resistance developing. As a consequence of newer antimicrobials, randomised controlled trial data [[41], [42], [43]], and the development of guidance including evidence-based options as outlined by the UK Clinical Pharmacy Association (UKCPA) Pharmacy Infection Network [44] and a report by the British Society for Antimicrobial Chemotherapy/Healthcare Infection Society/British Infection Association Joint Working Party [45], newer antimicrobials are starting to be used.

Fundamentally, antibiograms assist with antimicrobial stewardship and, in general, aminoglycosides are regarded as important empiric and oftentimes adjunctive agents in the antimicrobial armamentarium for the treatment of severe Gram negative infections. Akin to other studies [29], a higher susceptibility to amikacin (84%) compared to gentamicin (61.7%) was observed, which may support the case for preferential use of amikacin for possible combination therapy in CPE colonised patients with clinical sepsis. A concerning development in our study was emergent resistance to tigecycline; 87.2% of CPE isolates tested were susceptible in 2014 but this decreased to 64.3% in 2020. This bacteriostatic antimicrobial was oftentimes the first-line empiric choice in ULHG, administered alone or in combination to treat infections caused by CPE before the advent of newer β-lactam/β-lactamase inhibitor combinations launched for use in Ireland; ceftazidime/avibactam (January 2018) and meropenem/vaborbactam in December 2019. Indeed ceftazidime/avibactam has been increasingly prescribed for resistant Gram negative infections since 2018. Of the empiric ceftazidime/avibactam prescriptions, all 3 patients who received empiric treatment in 2018 were CPE positive (100%), 5 were CPE positive in 2019 (62.5%) and 10 were CPE positive on 2020 (52.6%). Yet, despite these prescriptions, only 2 patients had confirmed CPE infections.

Subsequent non-CPE Enterobacterales bacteraemia was assessed in this group and, interestingly, almost 95% of subjects did not receive empiric treatment effective against CPE despite developing signs of sepsis. It is not known whether the attending teams were aware of each patient's previous CPE status, and only 7 (37%) patients had an intervening negative CPE screen. Amongst those patients colonised with CPE who developed a non-CPE Enterobacterales blood stream infection, four (21%) died within 30 days of sepsis onset. Overall all causal mortality in those with CPE infections was 27% (8) and 55% (16) at 30 days and 90 days, respectively. However, a review into deaths of CPE hospitalised patients concluded that 8 patients died as an indirect result of CPE infection, which illustrates the complexity of understanding the contribution of CPE to causation of death. Those who have poor outcomes are oftentimes patients with severe underlying comorbidities and risk factors that put them at risk of CPE infection.

This study has several limitations. Firstly, it is a retrospective descriptive study of a group of hospitals in the mid-west area of Ireland that may not be generalizable to other centres, particularly given the unique infrastructural challenges in the main hospital site with six *Nightingale* style wards that potentially pose significant environmental risk factors for CPE acquisition that has not yet been fully elucidated. The lack of an electronic patient record impeded the analysis of certain surgical interventions due to missing notes within the patient charts. Genetic analysis of strains has not been completed for all isolates, yet would provide useful insight into transmission dynamics. Indeed analysis of the molecular characterization of circulating clones nationally is ongoing, and may provide further insights into spread between regions and hospitals. Although the general infection prevention and control measures implemented in ULHG have been described, their evolution over time and potential impact on the epidemiology of CPE in our region has not been explored. In particular, 2% chlorhexidine impregnated wash cloths (Clinicept® UK) are used in high risk areas to reduce the bioburden of organisms on the skin and thus risk of bacteraemia in our CPE colonised patients who have invasive devices [46].

In conclusion, although risk factors for acquisition of CPE have been studied previously, this report details the changing epidemiology of CPE colonisation and type of infections acquired by CPE positive patients in our region and it is the largest cohort of isolates described in Ireland to date. Our experience is generalisable to UK districts or university hospital setting given the commonality of IPC measures adopted in both countries [47]. Whilst it remains fundamental to optimise basic infection prevention and control practices such as hand & environmental hygiene as well as antimicrobial stewardship, one must ensure that specific CPE preventive interventions are implemented. These include screening for carriage, rapid diagnostics with timely isolation or cohorting of positive patients with identification and follow-up of contacts. Additional work is warranted to determine the role of the health-care environment, both ward accommodation as well as sanitary-ware in CPE transmission.

Prompt initiation of effective antimicrobial treatment is essential for CPE infections and further evidence is needed on judicious utilisation of newer anti-CPE drugs. Understanding the array of antimicrobial resistance mechanisms leads increasingly towards precision medicine for complex infections.

## Author contributions

N H O'Connell, S Gasior, B Minihan, S I Bhutta, J Powell and C Dunne collected and analysed the data. N H O'Connell and C Dunne participated in the writing of the manuscript. N O'Connell, S Gasior, J Powell B Slevin, S Barrett, L Power and C Dunne read and approved the final version of the manuscript.
