# Supplementary material for: Microbial epidemiology and clinical risk factors of carbapenemase-producing Enterobacterales amongst Irish patients from first detection in 2009 until 2020
Source: Infect Prev Pract. 2022 Jul 13;4(3):100230. doi: 10.1016/j.infpip.2022.100230 (PMC9352914; doi:10.1016/j.infpip.2022.100230)
Supplement: Multimedia component 1 [file mmc1.docx]

**Table 6. Confirmed CPE associated infections**

|  | **Source** | **Bacterial species and CPE enzyme** | **Patient**  **Age**  **Gender** | ***Empiric treatment*** | **Targeted antimicrobials** | **Outcome**  **30d** |
| --- | --- | --- | --- | --- | --- | --- |
| 1 | Urinary | KP  *KPC* | 94y  M | Targeted → | ^Tigecycline 50 mg 12h iv + Gentamicin 5mg/kg 24h iv 5d | Alive |
| 2 | Blood stream (*cSSTI source) | KP  *KPC* | 70y  M | Piperacillin/tazobactam4.5g 8h intravenous (iv) and Fluconazole 400mg 24h iv | Tigecycline 50mg 12h iv 23d given in combination with Gentamicin 5mg/kg 24h x 11d | Alive |
| 3 | Urinary | KP  *KPC* | 91Y  F | Coamoxiclav 1.2g 8h iv | Tigecycline 50mg 12h iv + Gentamicin 5mg/kg 24h iv x 7d -palliated | Died |
| 4 | Intra-abdominal | KP  *KPC* | 75yF | Piperacillin/tazobactam 4.5g 8h iv plus Metronidazole 500mg 8H iv and Gentamicin 5mg/kg 24h iv | Tigecycline 50 mg 12h iv x 10d + Gentamicin 5mg/kg 24h iv x 3d | Died |
| 5 | Intra-abdominal | KP  *KPC* | 51y  F | Meropenem 1g 8h iv | Tigecycline 50mg 12h iv plus Vancomycin 15mg/kg 12h iv x 15d. | Alive |
| 6 | Intra-abdominal | KP  *KPC* | 68yF | Meropenem 1g 8h iv Vancomycin 15mg/kg 12h iv and Fluconazole 400mg 24h iv | Tigecycline 50mg 12h iv 21d | Died |
| 7 | Respiratory | KP  *KPC* | 75y  F | Piperacillin / tazobactam 4.5g 8h iv | Tigecycline 50mg 12H iv + nebulised Colomycin 2mU 8h x 7d | Alive |
| 8 | cSSTI | CF  *KPC* | 80y  F | Piperacillin/tazobactam 4.5g 8h iv + Linezolid 600mg 12h iv + Metronidazole 500mg 8h iv | Tigecycline 50mg 12h iv + Piperacillin/tazobactam 4.5g 8h iv for 1d but palliated | Died |
| 9 | Blood –stream (abdominal) | KO  *KPC* | 56y  M | Meropenem 1g 8h iv and Gentamicin 5mg/kg 24h iv | Ciprofloxacin 400mg 12h iv 6d+ Gentamicin 5mg/kg for 3d, changed to Cotrimoxazole 2800mg 8h iv 6days reduced to 1400mg 8h iv for further 14d plus nebulised Colomycin 2 mU 8h x 29d. | Alive |
| 10 | Respiratory/ clinical sepsis | KP  *KPC* | 71y  F | Meropenem 1g 8h iv | Tigecycline 100mg 12h iv and Gentamicin 5mg/kg iv 24h x 14d | Alive |
| 11 | Respiratory | KP  KO  E.coli  *KPC* | 79y  F | Piperacillin/tazobactam 4.5g 8h iv | Tigecycline 50mg 12h iv x 48h then switched to Ciprofloxacin 400mg 12h iv and nebulised Colomycin 2mu 8h x 7d.  Palliated 2 months later | Alive |
| 12 | Urinary | KP  KPC | 84y  F | Meropenem 1g 8h iv | Tigecycline 50mg 12h iv + Gentamicin 5mg/kg 24h iv x 4d, then palliated | Died |
| 13 | Urinary | KP  *KPC* | 87y  M | Ciprofloxacin 500mg 12h orally (po) | Tigecycline 50mg 12h iv and Gentamicin 5mg/kg 24h iv x 7d | Alive |
| 14 | Intra-abdominal | KO  *KPC* | 37y  M | Piperacillin/tazobactam 4.5g 8h iv and Metronidazole 500mg 8h iv and Gentamicin 5mg/kg 24h iv | Tigecycline 50mg 12h iv x 72h then switched to Ciprofloxacin 500mg 12h po and Metronidazole 400mg 8h po x 7d | Alive |
| 15 | cSSTI | KP  *NDM* | 45y  F | Daptomycin 8mg/kg iv 48h | Tigecycline 50mg 12h iv x 15d, palliated | Alive |
| 16 | Blood stream (urinary source) | KP  *KPC* | 80y  F | Piperacillin/ tazobactam 4.5g 8h iv and gentamicin 5mg/kg iv | Tigecycline 100mg 12h iv plus Gentamicin 300mg 24h iv x 14d | Alive |
| 17 | Respiratory | KO  *KPC* | 76y  M | Cotrimoxazole 960mg 12h iv | Cotrimoxazole 960mg 12h iv 28d | Alive |
| 18 | Respiratory | KP  E. coli Serratia  *KPC* | 86yM | Piperacillin/tazobactam 4.5g 8h iv | Tigecycline 50mg 12h iv | Died |
| 19 | Intra-abdominal | KO  *KPC* | 64y  M | Piperacillin/tazobactam 4.5g 8h iv, vancomycin 15mg/kg 12h iv, Metronidazole 500mg 8h iv and Fluconazole 400mg 24h iv | Tigecycline 50mg 12h iv 18d (had also vancomycin 13d, ^$^Caspofungin 50mg 24h iv 15d and Gentamicin 5mg/kg 24h iv 6d course) | Alive |
| 20 | Intra-abdominal | KP  *KPC* | 56y  F | Meropenem 1g 8h iv and gentamicin 5mg/kg 24h iv | Ciprofloxacin 400mg 12h iv and Gentamicin 5mg/kg 7d; palliated | Died |
| 21 | Intra-abdominal | KP  *KPC* | 73y  M | Cotrimoxazole 960mg 12h iv + Amikacin 15mg/kg 24h | Tigecycline 50mg 12h iv x 23d | Alive |
| 22 | Intra-abdominal | CY  *KPC* | 81y  M | Ciprofloxacin 400mg 12h iv | Tigecycline 50mg 12h iv x 7d | Alive |
| 23 | cSSTI | E coli  *KPC* | 55y  F | Flucloxacillin 2g 6h iv | Ciprofloxacin 500mg 12h po + Amoxicillin 2g 8h iv and Metronidazole 500mg 8h iv 7 days | Alive |
| 24 | Bone & Joint | E coli  KP  EC  *KPC* | 65y  M | Linezolid 600mg 12h iv + Meropenem 1g 8h iv + Fluconazole 400mg 24h iv | Tigecycline 100mg 12h iv + Meropenem 2g 8h iv x 42d and Cotrimoxaozle 960mg 12h iv x 14d | Alive |
| 25 | cSSTI | KP  *KPC* | 77y  M | Piperacillin/tazobactam 4.5g 8h iv | Tigecycline 100mg 12h iv x 10d | Alive |
| 26 | cSSTI | CF  *KPC* | 81y  M | Ciprofloxacin 400mg 12h iv + Meropenem 1g 8h iv + Vancomycin 15mg/kg 12h iv | Tigecycline 100mg 12h iv + Amikacin 15mg/kg 24h iv but palliated | Died |
| 27 | Bone and Joint | KP  *OXA-48* | 46y  M | Daptomycin 10mg/kg 24h iv + Piperacillin/tazobactam 4.5g 8h iv | Ceftazidime/avibactam 2.5g 8h IV + Daptomycin 10mg/kg 24h IV x 80d | Alive |
| 28 | Blood (abdominal source) | KP  EC  E. coli  *KPC* | 55y  M | Piperacillin tazobactam 4.5g 8h iv + gentamicin 5mg/kg 24h iv | Ceftazidime/Avibactam,2g/0.5g 8h IV x 3d then Meropenem/Vaborbactam 5d, then Tigecycline 100mg 12h iv + Ciprofloxacin 400mg 12h iv 14d | Alive |
| 29 | Respiratory (clinical sepsis VRE colonised) | CF  KO  E.coli  *KPC* | 70y  M | Tigecycline 100 mg 12h iv + Daptomycin 10mg/kg 24h iv + metronidazole 500mg 8h iv | Ciprofloxacin 400mg 12 iv+ Amoxicillin 2g 4h iv x 10d | Alive |
| 30 | Intra-abdominal | KP  *KPC* | 25  F | Tigecycline 100mg loading then 50mg 12h iv plus Metronidazole 500mg 8h iv | Tigecycline 100mg loading then 50mg 12h iv plus Metronidazole 500mg 8h iv for 18d | Alive |

*complicated skin and soft tissue infection

^ loading dose of 100mg Tigecycline was given when prescribed in all cases

$ loading dose of 70mg Caspofungin was given when prescribed in all cases

*KP= K. pneumoniae, KO= K. oxytoca, CF= Citrobacter freundii, CY= Citrobacter youngae, EC= Enterobacter cloacae*

**Table 7. Non-CPE Enterobacterales bloodstream infections**

|  | Patient details  (age, gender, comorbidities) | Specimen and date of detection of CPE enzyme type | Follow-up screens | Non CPE BSI | Empiric treatment (Rx) | Targeted Rx | 30d outcome |
| --- | --- | --- | --- | --- | --- | --- | --- |
| 1 | 79y ^M  Cardiac, Renal  Pulmonary | Urine 9/1/16 KPC *K.pneumoniae* | 19/2/14 *NDT | *21/1/16 K.pneumoniae*  *E. coli* | Piperacillin/tazobactam 4.5g 8h iv | Palliative | Died |
| 2 | 82y M  Cardiac | Screen 26/3/14 KPC  *K.pneumoniae* | NIL | 31/7/14  *E. coli* | Piperacillin/tazobactam 4.5g 8h iv | Piperacillin/tazobactam 4.5g 8h iv x 10 d | Alive |
| 3 | 75y ^$^F  Malignancy | Screen 31/7/14 KPC  *Citrobacter freundii* | NIL | 29/12/14  *Proteus mirabilis* | Piperacillin/tazobactam 4.5g 8h iv | Piperacillin/tazobactam 4.5g 8h iv then switch to ciprofloxacin 500mg 12h po x 10d. Palliated. | Died |
| 4 | 65Y F Diabetic, Pulmonary, Renal | Screen 4/8/14 NDM *K. pneumoniae* | Nil | 10/8/15  *E. coli* | Piperacillin/tazobactam 4.5g 8h iv | Meropenem 1g 8h iv  Palliated | Died |
| 5 | 77y F Renal | Screen 18/11/14 KPC *K. pneumoniae* | 10/1/17 NDT | 9/1/18  *E. coli* | Piperacillin/tazobactam 4.5 g 8h iv | Piperacillin/tazobactam 4.5 g 8h iv x 10d | Alive |
| 6 | 86 M  Vasculopath, Pulmonary, Renal | Screen 11/12/14 KPC in *K. pneumoniae, E. coli & Serratia* sp. | 12/12/14 NDT | 16/1/15  *E. coli* | Piperacillin/tazobactam 4.5g 8h iv | Piperacillin/tazobactam 4.5g 8h iv x 7d | Alive |
| 7 | 47y F  Neurological, Vasculopath, Diabetic | Screen 16/1/15  OXA-48 *E. coli* | Nil sent | 5/5/15  *E. coli* | Meropenem 1g 8h iv | Meropenem 1g 8h iv x 7 days | Alive |
| 8 | 34y F  ^&^FMF | Screen 24/4/15 KPC *K. pneumoniae* | Nil sent | 1/2/16 *Pantoea agglomerans* fully sensitive | Piperacillin/tazobactam 4.g 8h iv | Piperacillin/tazobactam 4.g 8h iv changed to po Amoxicillin 1g 6h to complete 10d | Alive |
| 9 | 53y M  Diabetic | Screen 19/6/15 KPC *Citrobacter freundii* | Nil sent | 9/9/15 *Enterobacter cloacae* | Piperacillin/tazobactam 4.5g 6h iv | Meropenem 1g 8h iv 10d | Alive |
| 10 | 89y  M  Malignancy | Screen  14/8/15  *KPC K. oxytoca* | 4/9/15 NDT | 3/3/16  Extended spectrum producing Beta lactamase (ESBL) *E. coli* | Coamoxiclav 1.2g 8h iv and gentamicin 3mg/kg iv | Meropenem 1g 8h iv then de-escalated to Piperacillin/tazobactam 4.5g 8h iv to complete 10d | Alive |
| 11 | 85y  M  Biliary sepsis | Screen 25/8/15 KPC *K. oxytoca* | 15/3/17 NDT | 30/3/17  *E. coli* resistant to piperacillin/tazobactam | Piperacillin/tazobactam 4.5g 8h iv | Meropenem 1g 8h iv t/f to another centre for ERCP | Alive |
| 12 | 81y M  Prostatism | R/S 6/12/15 KPC *Citrobacter braakii* | 3/1/18  NDT | 28/1/18  ESBL *E. coli* | Piperacillin/tazobactam 4.5g 8h iv and Gentamicin 5mg/kg 24h iv | Piperacillin/tazobactam x 10d | Alive |
| 13 | 84y F Renal Cardiac | Screen  12/7/16  KPC *K. pneumoniae* | NDT between 08/18 and 07/19 | 27/10/19  E. coli resistant to piperacillin/tazobactam | Coamoxiclav 1.2g 8h iv and Gentamicin 5mg/kg 24 h iv | Piperacillin/tazobactam 4.5g 8h iv changed to Aztreonam 2g 8h iv to complete 7d | Alive |
| 14 | 81y M  Ulcerative colitis | Screen 28/7/16 KPC *Citrobacter youngae* | None sent | 3/8/16  *Serratia* sp. | Piperacillin/tazobactam 4.5 g 8h iv and Amikacin15mg/kg 24h iv | Piperacillin/tazobactam 4.5g 8h iv x 7d | Alive |
| 15 | 77y M  Vasculopath | Screen 13/6/17  KPC  *K. pneumoniae* | 27/10/17 NDT | 17/2/19  *E. coli* resistant to multiple agents including piperacillin/tazobactam | Piperacillin/tazobactam 4/5g 8h iv + Clarithromycin 500mg 12h iv + Gentamicin 5mg/kg 24h iv | Changed to Ceftazidime /avibactam 2.5g 8h iv + Fosfomycin 6g 4h iv but then de-escalated to Meropenem 1g 8h iv for 7 d | Alive |
| 16 | 68y M  Malignancy | Screen 13/7/17  KPC  *Citrobacter freundii* | 15/7/17 NDT | 24/4/18  *E. coli* resistant to amoxicillin and coamoxiclav | Piperacillin/tazobactam 4.5g 6h iv | Piperacillin/tazobactam 4.5g 6h iv x 7d | Died |
| 17 | 59y F  Hepatic | Screen  30/7/18 KPC *E. coli* | 4/9/18 NDT  28/11/18  NDT | 17/12/19  ESBL *E. coli* | Tigecycline 100mg 12h iv and Fosfomycin 6g 4h iv | Meropenem 1g 8h iv x 10d | Alive |
| 18 | 71y M  Hepatic  Renal | Screen 3/9/18  OXA- 48 *K. pneumoniae* | 3/2/20 NDT | 3/2/20 *E. coli* | Piperacillin/tazobactam 4.5g 8h iv | Ceftazidime/avibactam 2.5 g 8h iv and Gentamicin 5mg/kg iv changed to Ceftriaxone 2g 24 h iv 7d | Alive |
| 19 | 37y M  Malignancy | Screen 13/11/18 KPC Citrobacter sp. | None sent | 12/5/20  *E. coli* | Ceftriaxone 2g 24h iv | Amoxicillin 2g 8h iv 7d | Alive |

* NDT= Not detected

^ M= male ^$^F= female ^&^ FMF = Familial Mediterranean Fever
